# Brain atrophy in normal older adult links tooth loss and diet changes to future cognitive decline

**DOI:** 10.1038/s41514-024-00146-4

**Published:** 2024-03-22

**Authors:** Hiroyuki Nakamura, Moeko Noguchi-Shinohara, Mai Ishimiya-Jokaji, Yutaka Kobayashi, Mikana Isa, Kentaro Ide, Toshihiro Kawano, Shuichi Kawashiri, Kazuhiro Uchida, Yasuko Tatewaki, Yasuyuki Taki, Tomoyuki Ohara, Toshiharu Ninomiya, Kenjiro Ono

**Affiliations:** 1 Department of Oral and Maxillofacial Surgery, Graduate School of Medicine, University of the Ryukyus, Okinawa, Japan; 2 Department of Neurology, Kanazawa University Graduate School of Medical Sciences, Kanazawa, Japan; 3 Department of Oral and Maxillofacial Surgery, Kanazawa University Graduate School of Medical Sciences, Kanazawa, Japan; 4 Department of Health Promotion, School of Health and Nutrition Sciences, Nakamura-Gakuen University, Fukuoka, Japan; 5 Department of Nuclear Medicine and Radiology, Institute of Development, Aging and Cancer, Tohoku University, Sendai, Japan; 6 Department of Neuropsychiatry, Graduate School of Medical Sciences, Kyushu University, Fukuoka, Japan; 7 Department of Epidemiology and Public Health, Graduate School of Medical Sciences, Kyushu University, Fukuoka, Japan

**Keywords:** Dentistry, Cognitive ageing, Nutrition

## Abstract

Several studies have found associations between poor oral health, particularly tooth loss and cognitive decline. However, the specific brain regions affected by tooth loss and the probable causes remain unclear. We conducted a population-based longitudinal cohort study in Nakajima, Nanao City, Japan. Between 2016 and 2018, 2454 residents aged ≥60 participated, covering 92.9% of the local age demographics. This study used comprehensive approach by combining detailed dental examinations, dietary assessments, magnetic resonance imaging (MRI) analysis, and cognitive evaluations. Tooth loss, even in cognitively normal individuals, is associated with parahippocampal gyrus atrophy and increased WMH volume, both of which are characteristics of dementia. Tooth loss was associated with altered dietary patterns, notably a reduction in plant-based food intake and an increase in fatty, processed food intake. This study highlights a possible preventative pathway where oral health may play a significant role in preventing the early neuropathological shifts associated with dementia.

## Introduction

The world’s aging population is increasing; in fact, the number of people aged ≥60 years is estimated to reach 2.1 billion by 2050^1^. Together with this demographic change, age-related cognitive decline and dementia prevalence are dramatically increasing worldwide^2^. Tooth loss becomes more common with age, affecting more than 60% of elderly Japanese adults^3^. Natural tooth loss may affect chewing and alter dietary intake^4^. Emerging evidence suggests a potential link between oral health, diet, and cognitive function, highlighting the importance of a multidisciplinary approach to address the complex interplay between these factors^5–7^.

Several studies have established associations between oral health, particularly tooth loss, and cognitive decline. For instance, Naorungroj et al. found lower cognitive scores associated with edentulism, suggesting that past oral disease is a risk factor for cognitive decline^5^. Furthermore, Lee et al. reported that poor oral health and daily living activities were correlated with cognitive impairment^6^. Although evidence supports an association between oral health and cognitive decline, the specific brain regions affected by tooth loss and the potential mechanisms remain unclear. Additionally, the association of tooth loss on dietary patterns in cognitively normal individuals and its potential contribution to brain atrophy and cognitive decline requires further investigation.

The primary objective of this study was to explore the associations between oral functionality (tooth loss and denture usage), dietary consumption, cognitive decline, and dementia in a cohort of older adults Japanese individuals. Specifically, this study aimed to investigate the association of tooth loss on brain volume differences in mild cognitive impairment (MCI) and dementia and examine the dietary patterns associated with tooth loss in cognitively normal individuals as well as their potential implications for future cognitive decline. This study used a unique and comprehensive approach by combining detailed dental examinations, dietary assessments, magnetic resonance imaging (MRI) analysis, and cognitive evaluations in a large, well-characterized cohort of elderly Japanese individuals.

## Results

### Participant characteristics

In the present investigation, 919 participants were included in the analysis (409 men and 510 women, age, average [standard deviation, SD] 71.5 [7.0] years). MCI and dementia were determined using the clinical criteria described by Petersen et al.^8^ and the Third Diagnostics and Statistical Manual of Mental Disorders, Revised Edition (DSM-III-R)^9^. The characteristics of the participants according to their cognitive impairment are shown in Table 1. Among the 919 individuals, 163 belonged to the MCI group (17.7%) and 24 constituted the dementia group (2.6%). Significant differences were observed in age, sex, prevalence of diabetes mellitus, and duration of formal education across cognitive impairment statuses.

**Table 1:** Characteristics of the study population-based on cognitive impairment status

Variables	Total	Normal	MCI	Dementia	*F*-value	−Log10 (*p*-value)
*N* (%)	919 (100)	732 (79.6)	163 (17.7)	24 (2.6)		
Age, mean (SD), y	71.52 (7.07)	70.32 (6.49)	75.39 (7.23)	80.50 (4.76)	61.73	25.12
Women, %	55.5	56.7	44.7	80	7.98	3.43
Hypertension, %	53.6	52.5	58.8	52	3.00	1.30
Serum LDL-chol, mean (SD), mg/dL	115.25 (32.22)	115.66 (32.48)	114.85 (31.29)	106.12 (30.34)	1.03	0.44
Serum HDL-chol, mean (SD), mg/dL	60.56 (15.17)	61.04 (15.15)	59.37 (15.18)	54.91 (13.95)	2.52	1.09
Diabetes mellitus, %	16.4	15.3	19	32	3.26	1.41
Formal education, mean (SD), y	11.04 (2.31)	11.34 (2.23)	10.06 (2.35)	8.95 (1.42)	32.41	13.60

Values are expressed as mean (SD) unless otherwise stated.

### Association of tooth number or brain volume with cognitive impairment

Table 2 delineates the associations between tooth number, brain volume, and cognitive impairment, taking into account a range of variables including age, sex, educational level, hypertension, diabetes mellitus, and LDL and HDL cholesterol levels. The values are mean values accompanied by a 95% confidence interval. When examining the tooth number, both excluding and including dentures, no significant differences were observed across the groups with cognitively normal, MCI, and dementia conditions in the multivariable-adjusted models.

**Table 2:** Association of tooth number or brain volume with cognitive impairment

Variables	Normal	MCI	Dementia	*F*-value	−Log10 (*p*-value)
*N*	732	163	24		
1) Tooth number					
excluding dentures	14.84 (14.12–15.55)	14.41 (12.85–15.96)	13.44 (9.46–17.41)	0.30	0.13
including dentures	25.59 (25.24–25.93)	26.30 (25.55–27.06)	25.79 (23.87–27.71)	1.38	0.60
2) Brain volume to eTiv (%)					
Hippocampal	0.476 (0.472–0.480)	0.451 (0.443–0.460)^*^	0.420 (0.397–0.442)^*^	20.34	8.64
WMH	0.352 (0.325–0.379)	0.530 (0.471–0.589)^*^	0.612 (0.461–0.763)^*^	17.37	7.40
Entorhinal	0.226 (0.223–0.228)	0.220 (0.215–0.226)	0.188 (0.174–0.202)^*^	13.16	5.63
Parahippocampal	0.229 (0.227–0.232)	0.224 (0.219–0.229)	0.198 (0.185–0.212)*	10.27	4.41
Insula	0.829 (0.824–0.835)	0.802 (0.790–0.813)*	0.795 (0.764–0.825)	9.63	4.14
Lateralorbitofrontal	0.822 (0.816–0.828)	0.804 (0.792–0.817)*	0.783 (0.751–0.816)	5.00	2.16
Total brain	58.50 (58.27–58.72)	57.84 (57.35–58.34)	57.02 (55.77–58.28)	4.63	2.00
Posteriorcingulate	0.350 (0.347–0.353)	0.339 (0.332–0.346)*	0.347 (0.329–0.364)	4.11	1.78
Fusiform	1.271 (1.260–1.282)	1.263 (1.239–1.286)	1.189 (1.129–1.250)	3.42	1.48
Inferiortemporal	1.089 (1.080–1.098)	1.067 (1.047–1.086)	1.039 (0.988–1.089)*	3.42	1.48
Rostralanteriorcingulate	0.266 (0.263–0.269)	0.259 (0.252–0.267)	0.244 (0.226–0.263)	3.34	1.44
Caudalanteriorcingulate	0.223 (0.220–0.226)	0.218 (0.211–0.224)	0.203 (0.186–0.220)	3.11	1.34

Values are shown as mean values (95% confidence interval). In the multivariable-adjusted model, the values were adjusted for age, sex, educational level, hypertension, diabetes mellitus, and LDL and HDL cholesterol levels.

^*^Bonferroni-adjusted *p* values, *p* < 0.05 versus the cognitively unimpaired group. Omitted in the table are brain volumes with −log10 (*p* value) < 1.301. Each brain volumes to eTiv are expressed in % unless otherwise stated.

Analysis of brain volume relative to estimated total intracranial volume (eTiv) revealed more pronounced differences after applying a stringent bonferroni correction for multiple comparisons, with *p* values exceeding 0.05 threshold. Significant reductions were observed in the hippocampal volume in both the MCI (mean difference (MD) = −0.025%, 95% CI 0.443–0.460) and dementia (MD = −0.056%, 95% CI 0.390–0.442) groups compared with the cognitively normal (95% CI 0.472–0.480) group. Moreover, there was a significant increase in white matter hypointensity (WMH) volume in the MCI (MD = 0.178%, 95% CI 0.471–0.589) and dementia (MD = 0.26%, 95% CI 0.461–0.763) groups, with the dementia group showing the highest volume. In the MCI group compared with the cognitively normal group, the regional brain volumes of the insula (MD = −0.027%, 95% CI 0.790–0.813), lateral orbitofrontal cortex (MD = −0.018%, 95% CI 0.792–0.817), and posterior cingulate cortex (MD = −0.011%, 95% CI 0.332–0.346) were significantly reduced. In the dementia group, compared with the cognitively normal group, there were significant reductions in the regional brain volumes of the entorhinal cortex (MD = −0.038%, 95% CI 0.174–0.202), parahippocampal gyrus (MD = –0.031%, 95% CI 0.185–0.212), and inferior temporal gyrus (MD = −0.05%, 95% CI 0.988–1.089). Despite these significant findings, no significant volumetric differences were observed in the remaining 29 brain regions when comparing across the cognitive status groups.

### Association between tooth number and brain volume in cognitively normal individuals

Given that the results in Table 2 indicated no association between tooth number and cognitive impairment, the focus shifted to examining the relationship between tooth number and brain volume in cognitively normal individuals. Table 3 presents the detailed findings of this examination, showcasing the association between the number of residual teeth (excluding dentures) and regional brain volumes in cognitively unimpaired individuals after applying a stringent bonferroni correction with *p* values exceeding 0.05 threshold. The values are mean values accompanied by a 95% confidence interval, and have been adjusted for a variety of factors including age, sex, educational level, hypertension, diabetes mellitus, and LDL and HDL cholesterol levels in a multivariable model. In individuals with fewer than 10 residual teeth, there were significantly smaller volumes in the parahippocampal gyrus (MD = −0.008%, 95% CI 0.222–0.231), superior parietal cortex (MD = −0.043%, 95% CI 1.339–1.376), middle temporal gyrus (MD = −0.035%, 95% CI 1.193–1.227), lingual gyrus (MD = −0.02%, 95% CI 0.669–0.691), and banks of the superior temporal sulcus (bankssts) (MD = −0.009%, 95% CI 0.247–0.257) compared with individuals in the highest tertile with more than 24 teeth. A significantly greater volume of white matter hypointensities (WMHs) was noted (MD = 0.074%, 95% CI 0.335–0.414), highlighting a notable difference. The increased WMH volume and atrophy in parahippocampal gyrus, which were observed in individuals with less than 10 residual teeth, mirrored characteristics of dementia as outlined in Table 2. On the other hand, in individuals with 10–23 residual teeth (17.9 teeth left on average), there were no significant volume differences in the parahippocampal gyrus, superior parietal cortex, middle temporal gyrus, lingual gyrus, and bankssts compared with individuals in the highest tertile with over 24 teeth.

**Table 3:** Association of tooth number, excluding dentures, with brain volume in cognitively unimpaired individuals

Variables	Tooth number excluding dentures in cognitively unimpaired individuals			*F*-value	−Log10 (*p*-value)
	Tertile 1	Tertile 2	Tertile 3		
	less than 10	10–23	over 24		
*N*	252	251	229		
Tooth number	2.73 (2.35–3.12)	17.88 (17.5–18.26)	26.85 (26.44–27.25)		
Brain volume to eTiv (%)					
Parahippocampal※	0.227 (0.222–0.231)^*^	0.234 (0.230–0.238)	0.235 (0.231–0.240)	4.70	2.03
Superiorparietal	1.357 (1.339–1.376)*	1.378 (1.360–1.396)	1.400 (1.381–1.419)	4.63	2.00
Middletemporal	1.210 (1.193–1.227)*	1.241 (1.225–1.257)	1.245 (1.228–1.263)	4.50	1.94
WMH‡※	0.375 (0.335–0.414)*	0.298 (0.261–0.336)	0.301 (0.261–0.341)	4.45	1.92
Fusiform	1.085 (1.069–1.100)	1.116 (1.101–1.131)	1.096 (1.080–1.112)	4.18	1.80
Lingual	0.680 (0.669–0.691)*	0.690 (0.679–0.700)	0.700 (0.689–0.711)	3.16	1.37
Bankssts	0.252 (0.247–0.257)*	0.259 (0.254–0.263)	0.261 (0.256–0.266)	3.12	1.35
Parstriangularis	0.401 (0.394–0.408)	0.412 (0.406–0.419)	0.411 (0.403–0.418)	2.75	1.19
Total Brain	58.53 (58.14–58.92)	59.06 (58.68–59.43)	59.09 (58.69–59.49)	2.32	1.01
Superiortemporal	1.256 (1.239–1.272)	1.275 (1.259–1.291)	1.279 (1.262–1.296)	2.10	0.91
Parsorbitalis	0.241 (0.237–0.245)	0.247 (0.243–0.251)	0.247 (0.243–0.251)	2.06	0.89
Precentral	1.419 (1.400–1.437)	1.439 (1.421–1.457)	1.446 (1.427–1.465)	2.05	0.89
Rostralmiddlefrontal	1.625 (1.605–1.646)	1.645 (1.626–1.665)	1.656 (1.634–1.677)	2.00	0.86
Supramarginal	1.113 (1.098–1.129)	1.134 (1.119–1.149)	1.128 (1.113–1.144)	1.88	0.82
Posteriorcingulate‡	0.350 (0.344–0.355)	0.351 (0.346–0.357)	0.357 (0.351–0.362)	1.58	0.68
Frontalpole	0.106 (0.103–0.108)	0.108 (0.105–0.110)	0.109 (0.106–0.112)	1.41	0.61
Medialorbitofrontal	0.606 (0.598–0.614)	0.611 (0.603–0.618)	0.615 (0.607–0.623)	1.15	0.50
Insula‡	0.833 (0.823–0.843)	0.834 (0.824–0.843)	0.824 (0.814–0.834)	1.15	0.50
Entorhinal※	0.225 (0.220–0.229)	0.229 (0.225–0.233)	0.228 (0.224–0.233)	1.13	0.49
Pericalcarine	0.240 (0.235–0.245)	0.242 (0.238–0.247)	0.245 (0.240–0.250)	1.08	0.47
Inferiorparietal	1.453 (1.433–1.472)	1.468 (1.449–1.486)	1.451 (1.431–1.470)	0.97	0.42
Precuneus	1.016 (1.004–1.029)	1.022 (1.010–1.034)	1.029 (1.016–1.042)	0.95	0.41
Rostralanteriorcingulate	0.264 (0.258–0.269)	0.269 (0.263–0.274)	0.268 (0.262–0.274)	0.94	0.41
Temporalpole	0.289 (0.284–0.294)	0.286 (0.281–0.290)	0.283 (0.278–0.289)	0.93	0.40
Inferiortemporal※	1.288 (1.268–1.307)	1.272 (1.253–1.290)	1.286 (1.266–1.306)	0.88	0.38
Superiorfrontal	2.364 (2.338–2.391)	2.383 (2.357–2.409)	2.387 (2.360–2.415)	0.74	0.32
Caudalanteriorcingulate	0.222 (0.216–0.227)	0.225 (0.220–0.230)	0.226 (0.221–0.232)	0.65	0.28
Parsopercularis	0.450 (0.442–0.458)	0.452 (0.444–0.459)	0.456 (0.448–0.464)	0.65	0.28
Postcentral	1.019 (1.005–1.033)	1.014 (1.000–1.027)	1.024 (1.010–1.039)	0.54	0.23
Lateralorbitofrontal‡	0.823 (0.812–0.833)	0.829 (0.819–0.839)	0.830 (0.819–0.841)	0.49	0.21
Lateraloccipital	1.181 (1.164–1.198)	1.191 (1.175–1.207)	1.191 (1.174–1.208)	0.46	0.20
Caudalmiddlefrontal	0.625 (0.614–0.637)	0.619 (0.608–0.630)	0.625 (0.613–0.637)	0.42	0.18
Hippocampal‡※	0.480 (0.473–0.487)	0.484 (0.477–0.491)	0.483 (0.475–0.490)	0.38	0.17
Isthmuscingulate	0.284 (0.279–0.289)	0.286 (0.281–0.291)	0.287 (0.282–0.292)	0.27	0.12
Transversetemporal	0.109 (0.106–0.111)	0.108 (0.106–0.110)	0.109 (0.107–0.111)	0.26	0.11
Cuneus	0.307 (0.302–0.312)	0.306 (0.302–0.311)	0.309 (0.304–0.314)	0.21	0.09
Paracentral	0.383 (0.377–0.390)	0.384 (0.378–0.390)	0.384 (0.378–0.390)	0.01	0.01

Values are shown as mean values (95% confidence interval). In the multivariable-adjusted model, the values were adjusted for age, sex, educational level, hypertension, diabetes mellitus, and LDL and HDL cholesterol levels.

*Bonferroni-adjusted *p* values, *p* < 0.05 versus the over 24 tooth number group. Brain volume was significantly changed in individuals with ‡ MCI or ※dementia in Table 2, *p* < 0.05 versus the cognitively unimpaired group. Each brain volumes to eTiv are expressed in % unless otherwise stated.

Table 3, shows significant alterations in brain volume among individuals with fewer than 10 natural teeth. This intriguing finding prompted a deeper investigation into the potential influence of denture usage on brain volume in individuals with fewer than 10 natural teeth. Table 4 indicated that no differences in brain volumes were observed between denture users and nonusers within the group of individuals having fewer than 10 natural teeth. Most participants within this category of 10 natural teeth were denture users.

**Table 4:** Association of denture usage in cognitively unimpaired individuals with brain volume

Variables	Less than 10 tooth excluding dentures in cognitively unimpaired individuals			
	Median 1	Median 2	*F*-value	−Log10 (*p*-value)
	No denture usage	Denture usage		
*N*	16	236		
Tooth number	2.25 (0.73–3.76)	2.38 (1.98–2.78)		
Brain volume to eTiv (%)				
Bankssts	0.234 (0.216–0.252)	0.249 (0.245–0.254)	2.69	0.99
Temporalpole	0.273 (0.252–0.294)	0.289 (0.284–0.295)	2.26	0.87
Parsopercularis	0.463 (0.433–0.494)	0.440 (0.432–0.449)	2.05	0.81
Fusiform	1.033 (0.970–1.096)	1.065 (1.048–1.081)	0.92	0.47
Postcentral	1.031 (0.972–1.089)	1.002 (0.986–1.017)	0.88	0.46
Superiortemporal	1.200 (1.136–1.265)	1.232 (1.215–1.249)	0.88	0.46
Parstriangularis	0.384 (0.357–0.411)	0.397 (0.390–0.405)	0.86	0.45
Inferiorparietal	1.388 (1.306–1.469)	1.426 (1.405–1.448)	0.82	0.44
Insula‡	0.814 (0.771–0.856)	0.833 (0.822–0.844)	0.75	0.41
Rostralmiddlefrontal	1.639 (1.548–1.730)	1.601 (1.576–1.625)	0.63	0.37
Lingual	0.684 (0.643–0.725)	0.667 (0.656–0.678)	0.62	0.36
Parsorbitalis	0.232 (0.216–0.248)	0.238 (0.234–0.242)	0.53	0.33
Total brain	58.19 (56.32–60.06)	57.51 (57.02–58.01)	0.47	0.31
Lateralorbitofrontal‡	0.798 (0.754–0.841)	0.813 (0.802–0.825)	0.45	0.30
Caudalmiddlefrontal	0.600 (0.555–0.646)	0.616 (0.604–0.628)	0.44	0.29
Inferiortemporal※	1.282 (1.202–1.362)	1.258 (1.237–1.279)	0.33	0.25
Precuneus	0.987 (0.936–1.038)	1.002 (0.989–1.016)	0.33	0.25
Hippocampal‡※	0.459 (0.431–0.488)	0.467 (0.459–0.474)	0.27	0.22
Isthmuscingulate	0.286 (0.262–0.311)	0.280 (0.274–0.286)	0.24	0.21
Paracentral	0.383 (0.360–0.407)	0.378 (0.372–0.384)	0.21	0.19
Transversetemporal	0.109 (0.099–0.118)	0.106 (0.104–0.109)	0.21	0.19
Caudalanteriorcingulate	0.224 (0.201–0.247)	0.219 (0.213–0.225)	0.17	0.17
Entorhinal※	0.218 (0.199–0.236)	0.222 (0.217–0.227)	0.18	0.17
Precentral	1.379 (1.302–1.456)	1.396 (1.376–1.416)	0.18	0.17
Supramarginal	1.107 (1.049–1.164)	1.094 (1.079–1.109)	0.17	0.17
Cuneus	0.308 (0.288–0.328)	0.304 (0.298–0.309)	0.15	0.16
Rostralanteriorcingulate	0.266 (0.245–0.287)	0.262 (0.256–0.267)	0.14	0.15
Parahippocampal※	0.224 (0.207–0.242)	0.221 (0.216–0.226)	0.11	0.13
Frontalpole	0.108 (0.097–0.118)	0.106 (0.103–0.109)	0.10	0.12
Superiorparietal	1.329 (1.250–1.408)	1.339 (1.319–1.360)	0.06	0.10
Middletemporal	1.194 (1.126–1.263)	1.186 (1.168–1.204)	0.06	0.09
Lateraloccipital	1.161 (1.094–1.229)	1.157 (1.139–1.175)	0.02	0.05
Pericalcarine	0.240 (0.220–0.260)	0.239 (0.233–0.244)	0.02	0.05
Posteriorcingulate‡	0.346 (0.322–0.369)	0.344 (0.338–0.350)	0.02	0.05
WMH‡※	0.436 (0.248–0.625)	0.431 (0.381–0.481)	0.00	0.02
Medialorbitofrontal	0.602 (0.568–0.636)	0.603 (0.594–0.612)	0.00	0.02
Superiorfrontal	2.330 (2.214–2.446)	2.334 (2.303–2.365)	0.00	0.02

Values are shown as mean values (95% confidence interval). In the multivariable-adjusted model, the values were adjusted for age, sex, educational level, hypertension, diabetes mellitus, and LDL and HDL cholesterol levels.

^*^Bonferroni-adjusted *p* values, *p* < 0.05 versus the denture usage group. Brain volume was significantly changed in individuals with ‡MCI or ※dementia as shown in Table 2, *p* < 0.05 versus the cognitively unimpaired group. Each brain volumes to eTiv are expressed in % unless otherwise stated.

### Dietary patterns associated with tooth loss in cognitively normal individuals

Table 5 elucidates the relationship between the number of residual teeth excluding dentures and nutrient intake in cognitively unimpaired individuals. The analysis categorizes individuals into three groups based on their residual tooth count: less than 10, between 10 and 23, and over 24 teeth. The nutrient intake is then analyzed across these groups, with the findings adjusted for age and sex, and presented with a 95% confidence interval. The data reveals a detailed picture of how tooth loss can affect dietary habits. Individuals with less than 10 residual teeth demonstrated a significantly reduced intake of several nutrients compared with those with more than 24 teeth after applying a stringent bonferroni correction with *p* values exceeding 0.05 threshold. This group consumed less dietary fiber, manganese, copper, iron, vitamin K, molybdenum, and vitamin C. The decrease in the intake of these essential nutrients underscores the potential nutritional challenges faced by individuals with a higher degree of tooth loss. The study also identified nutrients whose intake increased in the group with the fewest residual teeth. These individuals had a higher consumption of α-tocopherol, energy, γ-tocopherol, α-linolenic acid, and arachidic acid than those with over 24 residual teeth. Moreover, the findings indicate that individuals in the middle tertile, with 10–23 residual teeth (17.9 teeth left on average), generally maintained nutrient intake levels that were more comparable to those in the lowest tertile, rather than those with over 24 teeth.

**Table 5:** Association of tooth number, excluding dentures, with nutrients intake in cognitively unimpaired individuals

	Tooth number excluding dentures in cognitively unimpaired individuals				
Variables	Tertile 1	Tertile 2	Tertile 3	*F*-value	-Log10 (*p*-value)
	less than 10	11 to 23	over 24		
*N*	252	251	229		
Tooth number	2.73 (2.35–3.12)	17.88 (17.5–18.26)	26.85 (26.44–27.25)		
1) Decreases with tooth loss					
Dietary fiber, g	12.25 (11.60–12.90)*	13.78 (13.15–14.41)	13.72 (13.04–14.39)	6.47	2.79
Manganese, mg	3.37 (3.20–3.54)*	3.74 (3.58–3.90)	3.70 (3.53–3.88)	5.34	2.30
Copper, mg	1.11 (1.07–1.15)*	1.21 (1.17–1.25)	1.17 (1.12–1.21)	4.93	2.13
Iron, mg	7.87 (7.53–8.21)*	8.63 (8.30–8.96)	8.28 (7.93–8.64)	4.91	2.12
Vitamin K, ug	252.50 (235.20–269.81)*	285.66 (268.93–302.38)	282.23 (264.36–300.10)	4.10	1.77
Molybdenum, ug	221.70 (212.96–230.44)*	238.87 (230.42–247.32)	226.95 (217.92–235.97)	4.05	1.75
Surplus ammonia, mg	16.70 (15.41–18.00)	18.87 (17.61–20.12)	19.00 (17.66–20.34)	3.57	1.54
Vitamin C, mg	101.89 (95.20–108.58)*	110.32 (103.85–116.79)	115.06 (108.15–121.97)	3.51	1.52
Iodine, ug	1866.19 (1665.76–2066.62)	2206.89 (2013.17–2400.62)	2204.65 (1997.66–2411.64)	3.49	1.51
Ammonia, mg	1184.05 (1135.73–1232.36)*	1268.01 (1221.30–1314.71)	1208.97 (1159.07–1258.87)	3.19	1.38
Folic acid, ug	338.04 (318.51–357.57)	370.51 (351.64–389.39)	368.19 (348.02–388.36)	3.16	1.37
Nitrate ion, g	0.20 (0.18–0.21)	0.22 (0.20–0.24)	0.22 (0.21–0.24)	3.03	1.31
2) Increases with tooth loss					
β-tocopherol, mg	0.33 (0.31–0.35)*	0.34 (0.32–0.35)*	0.31 (0.29–0.32)	3.59	1.55
Energy, kcal	1818.00 (1765.39–1870.61)*	1890.35 (1839.50–1941.20)*	1796.89 (1742.56–1851.22)	3.50	1.51
δ-tocopherol, mg	3.43 (3.24–3.62)*	3.63 (3.45–3.81)*	3.28 (3.09–3.47)	3.42	1.48
α–linolenic acid, mg	1462.34 (1385.20–1539.48)*	1474.03 (1399.47–1548.59)*	1340.67 (1261.00–1420.33)	3.39	1.46
γ-tocopherol, mg	11.81 (11.17–12.45)*	12.38 (11.76–13.00)*	11.22 (10.56–11.88)	3.21	1.39
Arachidic acid, mg	177.44 (168.85–186.04)*	184.55 (176.24–192.85)*	169.23 (160.36–178.11)	3.11	1.34

Values are shown as mean values (95% confidence interval). In the multivariable-adjusted model, the values were adjusted for age and sex.

*Bonferroni-adjusted *p* values, *p* < 0.05 versus the over 24 tooth number group. Omitted in the figure are nutrients intake with −log10 (*p* value) < 1.301.

Table 6 shows two primary dietary patterns that represent the variations in nutrient intake linked to tooth loss, as determined by reduced rank regression analysis. The first dietary pattern predominantly governs the nutrients that decline with tooth loss. This pattern elucidates a substantial part of the nutrient variation, accounting for 70.4% of the total explained variation. It manifests strong positive correlations with the intake of several nutrients, including dietary fiber, manganese, copper, iron, vitamin K, molybdenum, vitamin C, and ammonia, indicating that a higher score in this dietary pattern is associated with increased intake of these nutrients. This pattern also extends its influence to food and beverage items, explaining 4.9% of their variation, thereby offering a comprehensive view of the dietary shifts accompanying tooth loss. Conversely, the second dietary pattern emerges as a significant influencer in the context of nutrients that experience an uptick with tooth loss, explaining a remarkable 78.0% of the variation in these nutrients. This pattern harbors positive correlations with β-tocopherol, energy, δ-tocopherol, α-linolenic acid, γ-tocopherol, and arachidic acid, suggesting that individuals with a higher adherence to this dietary pattern tend to have an increased intake of these nutrients. Moreover, this pattern elucidates 3.6% of the variation in food and beverage items, painting a detailed picture of the dietary adaptations that occur with tooth loss.

**Table 6:** Percentage of variation in nutrient intake explained via derived dietary patterns

	DP1	Dietary patterns		DP4	Total explained variation
		DP2	DP3		
1) Decreased nutrients with tooth loss < 10					
Explained variation in nutrients, %	70.45	10.91	8.19	5.75	95.32
Explained variation in food and beverage items, %	4.93	1.34	2.18	2.02	10.49
Pearson correlation coefficients					
Dietary fiber	0.885**	0.023	−0.370**	−0.010	
Manganese	0.700**	0.520**	0.463**	0.041	
Copper	0.956**	−0.125**	0.142**	−0.012	
Iron	0.935**	0.022	0.140**	−0.209**	
Vitamin K	0.842**	−0.224**	−0.254**	0.353**	
Molybdenum	0.777**	−0.457**	0.336**	0.222**	
Vitamin C	0.758**	0.542**	−0.267**	0.141**	
Ammonia	0.829**	−0.184**	−0.129**	−0.470**	
2) Increased nutrients with tooth loss < 10					
Explained variation in nutrients, %	78.02	12.02	4.98	2.51	97.53
Explained variation in food and beverage items, %	3.57	2.03	1.45	1.43	8.51
Pearson correlation coefficients					
β-tocopherol	0.494**	0.289**	−0.096**	−0.076	
Energy	0.576**	0.419**	−0.007	0.029	
δ-tocopherol	0.631**	0.384**	−0.131**	−0.113**	
α-linolenic acid	0.487**	0.206**	−0.063	−0.042	
γ-tocopherol	0.555**	0.251**	−0.071	−0.066	
Arachidic acid	0.490**	0.230**	−0.002	0.024	

Dietary patterns were derived by reduced rank regression analysis. Pearson correlation coefficients showing associations between dietary pattern scores and nutrient intake.

^**^p values < 0.001.

The data in Table 7 show that individuals with a significant amount of tooth loss, defined as having fewer than 10 teeth, tend to consume less nutrients, primarily found in plant-based foods. This category encompasses a wide variety of items including vegetables—both high and not high in beta-carotene—fruits, soy derivatives such as tofu and natto, mushrooms, seaweed, green tea, and potatoes. These foods, known for their dense nutrient profile, are also characterized by a texture that demands substantial chewing, a task that becomes increasingly arduous with diminishing teeth count. The correlations presented in the table vividly illustrate this trend, with significant positive correlations observed between these food groups and a range of nutrients, including dietary fiber, manganese, copper, iron, vitamin K, molybdenum, vitamin C, and ammonia, thereby underscoring the nutrient-rich nature of these foods and the consequent nutrient loss associated with reduced intake. In contrast, the landscape of dietary patterns shifts dramatically as we move to foods that are more accommodating to a compromised dentition. The data unveils a surge in the intake of nutrients housed in softer, high-calorie, and often processed foods, a category that houses items such as blended oils, various fats, a spectrum of meats including beef, pork, and chicken, alongside eggs and dressings such as the French dressing. These foods, characterized by their softer texture and higher calorie content, present a more manageable option for individuals with severe tooth loss, facilitating easier consumption. The table elucidates this trend through notable positive correlations between these food groups and nutrients such as β-tocopherol, energy, δ-tocopherol, α-linolenic acid, γ-tocopherol, and arachidic acid, painting a picture of a dietary pattern leaning heavily towards increased intake of these nutrients.

**Table 7:** Factor loadings of food groups associated with dietary pattern 1 and correlation coefficients between food groups and nutrients intake

Food groups	Factor loading (DP1)			Correlations between food groupes and response variables					
1) Decreases with tooth loss < 10		Dietary fiber	Manganese	Copper	Iron	Vitamin K	Molybdenum	Vitamin C	Ammonia
Vegetables not high in beta-carotene	0.234	0.753**	0.362**	0.547**	0.468**	0.649**	0.337**	0.680**	0.424**
Vegetables high in beta-carotene	0.233	0.704**	0.351**	0.520**	0.501**	0.699**	0.284**	0.709**	0.441**
Tofu	0.203	0.416**	0.342**	0.564**	0.550**	0.431**	0.505**	0.344**	0.510**
Natto (soybeans)	0.202	0.418**	0.155**	0.529**	0.420**	0.789**	0.729**	0.183**	0.402**
Mushrooms	0.195	0.612**	0.282**	0.492**	0.478**	0.441**	0.292**	0.446**	0.464**
All seafood	0.194	0.373**	0.267**	0.530**	0.565**	0.375**	0.308**	0.321**	0.723**
Seaweed	0.189	0.567**	0.299**	0.455**	0.476**	0.452**	0.324**	0.411**	0.406**
Potato	0.172	0.476**	0.296**	0.438**	0.401**	0.379**	0.268**	0.456**	0.387**
Other soybeans	0.170	0.452**	0.280**	0.436**	0.460**	0.364**	0.289**	0.353**	0.422**
All meat	0.161	0.325**	0.248**	0.427**	0.541**	0.322**	0.257**	0.281**	0.480**
Green fish	0.160	0.325**	0.214**	0.431**	0.438**	0.360**	0.266**	0.264**	0.564**
Fruits	0.153	0.510**	0.220**	0.356**	0.295**	0.299**	0.119**	0.619**	0.350**
White fish	0.146	0.283**	0.218**	0.392**	0.395**	0.274**	0.227**	0.263**	0.559**
Chicken	0.142	0.287**	0.224**	0.378**	0.434**	0.312**	0.257**	0.247**	0.415**
Green tea	0.141	0.169**	0.860**	0.274**	0.445**	0.143**	0.102**	0.522**	0.143**
2) Increases with tooth loss < 10		β-tocopherol	Energy	δ-tocopherol	α-linolenic acid	γ-tocopherol	Arachidic acid		
Blended oil	0.310	0.629**	0.370**	0.518**	0.805**	0.759**	0.643**		
Fats	0.303	0.659**	0.373**	0.474**	0.742**	0.733**	0.660**		
Tofu	0.237	0.522**	0.350**	0.651**	0.437**	0.510**	0.393**		
French dressing	0.226	0.489**	0.185**	0.363**	0.625**	0.578**	0.458**		
All meat	0.204	0.320**	0.557**	0.317**	0.363**	0.347**	0.652**		
Soybeans	0.178	0.355**	0.315**	0.415**	0.374**	0.376**	0.328**		
All seafood	0.168	0.240**	0.580**	0.281**	0.281**	0.266**	0.486**		
Natto (soybeans)	0.155	0.353**	0.202**	0.496**	0.282**	0.346**	0.182**		
Chicken eggs	0.151	0.279**	0.349**	0.290**	0.311**	0.322**	0.309**		
Mushrooms	0.148	0.267**	0.321**	0.332**	0.287**	0.320**	0.290**		
Beef	0.146	0.210**	0.368**	0.179**	0.240**	0.227**	0.605**		
Chicken	0.146	0.252**	0.394**	0.278**	0.269**	0.261**	0.363**		
Pork	0.144	0.221**	0.400**	0.218**	0.252**	0.244**	0.462**		

^**^p value < 0.001. Omitted in the figure are factor loadings < 0.140.

## Discussion

This study explored the relationships among oral functionality, dietary consumption, cognitive decline, and dementia in a cohort of 919 elderly Japanese individuals. Unlike previous reports^5–7^, the results did not show a significant association between tooth loss and cognitive impairment, regardless of whether dentures were used or not. This discrepancy could potentially stem from differences in study populations, adjustments for confounders, and methods used to determine cognitive status and tooth loss. However, there were significant reductions in hippocampal volume and increases in WMHs volume in the groups with MCI and dementia compared with the cognitively normal group. In the MCI group, the regional brain volumes of the insula, lateral orbitofrontal cortex, and the posterior cingulate cortex were significantly reduced. In the dementia group, there were significant reductions in the regional brain volumes of the entorhinal cortex, parahippocampal gyrus, and inferior temporal gyrus.

Moreover, cognitively normal individuals with less than 10 teeth had significantly smaller volumes of the parahippocampal gyrus, superior parietal cortex, middle temporal gyrus, lingual cortex, and bankssts and a greater WMH volume than those with the most residual teeth (highest tertile). This suggests that tooth loss, even in cognitively normal individuals, is associated with atrophy of the parahippocampal gyrus and greater WMH volume, which are both characteristics of dementia. This result also led us to question the potential role of dentures in the fewer-than-10-teeth category. However, the use of dentures did not exhibit a discernible influence on brain volumes. Interestingly, most participants in this category were denture users. This suggests the possibility that the brain volume changes observed in the parahippocampal gyrus and WMH may occur even with denture usage. This means that once an individual’s natural tooth count drops below a threshold, in this case, 10 teeth, the introduction of dentures might not be sufficient to counteract the associated changes in brain volume. The importance of maintaining more than 10 natural teeth is evident.

One of the possible aspects of this relationship is the role of the periodontal ligaments. Natural teeth possess these ligaments, which are connected to the trigeminal nerve, playing a pivotal role in transmitting sensory information to the brain^10^. The loss of this vital connection, due to tooth loss, might lead to reduced stimulation of the brain, potentially affecting regions responsible for processing this sensory information. This could explain the observed decrease in brain volume in certain areas. Supporting this theory, research has shown that molar tooth loss can lead to hippocampus-dependent spatial memory impairment^11^. Specifically, mice with removed molars exhibited declines in hippocampal brain-derived neurotrophic factor levels and spatial memory^11^. Another study found that aged molarless mice showed a significantly reduced learning ability in a water maze test compared with age-matched control mice. Immunohistochemical analysis showed that the molarless condition enhanced the age-dependent increase in the density and hypertrophy of GFAP-labeled astrocytes in the CA1 region of the hippocampus. These effects increased the longer the molarless condition persisted, suggesting that impairment of spatial memory and changes in astroglial responsiveness occur following the loss of molar teeth in aged mice^12^. Thus, dentures and implants, lacking these periodontal ligaments, might not provide the same level of sensory stimulation to the brain. However, it’s essential to approach these findings with caution. The small sample size of non-denture users (only 16 individuals) in our study suggests a need for further research with a larger cohort to validate these observations. This would offer a more comprehensive understanding of the intricate relationship between dental health and brain volume, paving the way for potential interventions to mitigate cognitive decline in older adults.

The observed correlation between tooth loss and atrophy of the parahippocampal gyrus in our study can be attributed to a series of intertwined physiological and cognitive mechanisms. Central to this association is the intricate functionality of the parahippocampal gyrus and its neighboring structure, the hippocampus. These neural regions are not only fundamental in encoding and retrieving taste memories and play a pivotal role in assimilating and sustaining broader episodic memories^13^. As an individual’s count of natural teeth diminishes, there’s a plausible decline in their capacity to fully discern and appreciate diverse flavors in food. This compromised taste perception can lead to a diminished gastronomic experience, making meals less palatable and consequently less enjoyable. Delightful meals enriched with flavors and shared emotions significantly contribute to the creation and preservation of episodic memories. These memories encapsulate not only the sensory experience of the food but also the ambiance, the company, and the myriad emotions intertwined with the moment. A decline in taste perception, stemming from tooth loss, can thus have cascaded effects on this intricate memory tapestry, leading to less vivid episodic memories. Over an extended period, this diminished sensory and emotional engagement could result in decreased stimulation of the parahippocampal gyrus. This lack of neural activation may be a contributing factor to its atrophy. They hint at the possibility that maintaining dental health might extend beyond aesthetics and functionality, potentially playing a role in preserving the neural structures integral to our memories and cognitive well-being.

In addition, the study found that tooth loss was associated with altered dietary patterns, specifically a reduction in the intake of plant-based foods, including vegetables, fruits, soy foods, mushrooms, seaweed, green tea, and potatoes, and an increase in the intake of fatty, processed foods. These dietary patterns were correlated with the severity of tooth loss and may contribute to greater WMH volume and cognitive decline through mechanisms such as oxidative stress, inflammation, and vascular dysfunction. Moreover, the findings indicate that individuals in the middle tertile, with 10 to 23 residual teeth (17.9 teeth left on average), generally maintained nutrient intake levels that were more comparable to those in the lowest tertile, rather than those with over 24 teeth. This observation hints at a gradient effect where the association on nutrient intake becomes more pronounced when there are fewer than 10 natural teeth. The findings emphasize the importance of maintaining at least 10 or more than 17.9 natural teeth in preserving both nutritional status and brain health as we age. The previous research indicates that retaining teeth is associated with various lifestyle factors, including smoking, alcohol intake, and potentially oral hygiene, which could indirectly relate to socioeconomic status (such as poverty). It’s clear that maintaining oral health is not only about dental care but also involves broader health and lifestyle considerations^14,15^. While previous studies have suggested a link between oral health and cognitive decline^16–19^, our study provides more detailed insights into the specific WMH regions affected and the potential mechanisms involved.

There are several limitations to this study. First, the cross-sectional design of the study did not allow us to establish causality between tooth loss, brain atrophy, and cognitive decline. Longitudinal studies are required to confirm the causal relationships between these variables. Second, the study population consisted of elderly Japanese individuals, and the results may not be generalizable to other populations. Third, the study did not assess other potential confounding factors, such as oral health status, periodontal disease, and use of dental prosthetics, which could have influenced the results. Finally, dietary intake was assessed using a self-administered diet history questionnaire, which may be subject to recall bias.

In conclusion, this study unveils a potentially promising finding that even in cognitively normal individuals, tooth loss may be intricately linked to brain atrophy and shifts in dietary patterns, potentially laying a foundation for future cognitive decline and dementia. This is especially significant in the context of the established understanding that neuropathological alterations, including changes in brain volume, precede the clinical symptoms of Alzheimer’s disease (AD) by many years^20–23^. This highlights a potential preventative pathway in which attention to oral health may be a significant step in preventing the early neuropathological shifts associated with AD. This revelation holds substantial implications for clinicians, policymakers, and patients alike, underscoring the imperative of sustaining good oral health and a balanced diet as proactive measures to forestall cognitive decline and dementia.

## Methods

### Study population

This report describes an ongoing study aiming to investigate cognitive deterioration in older adults Japanese individuals. Dementia screening was conducted on a population-based, longitudinal cohort of individuals within a specific age range. This study was conducted in Nakajima, situated in Nanao City within the Ishikawa Prefecture, Japan, and the research methodology has been previously described^16,24^. From 2016 to 2018, 2454 inhabitants aged ≥60 (representing 92.9% of the total demographics within this age bracket) participated in the study. We excluded individuals who had no brain MRI examinations (*n* = 1159), no dental examinations (*n* = 302), hemorrhagic and/or ischemic stroke lesions on MRI regardless of the presence or absence of neurological symptoms (*n* = 69), and no dietary history evaluations (*n* = 5). Stroke lesions (hemorrhagic and/or ischemic) were identified by two trained neuroradiologists blinded to the clinical information. Finally, data from 919 individuals were included in the analysis.

### Ethics statements

This study complied with the principles outlined in the Declaration of Helsinki, and all methodologies were approved by the Kanazawa University Medical Ethics Review Committee (Approval Number 2185) and Ryukyu University Medical Ethics Review Committee (Approval Number 2153). We obtained written informed consent from all participants.

### Cognitive status

Diagnosis of dementia was based on the guidelines of the Diagnostic and Statistical Manual of Mental Disorders, third edition, revised (DSM-III-R)^9^, whereas diagnosis of MCI was established according to the International Working Group on general criteria for MCI^25^, which state that (1) persons should be judged as abnormal using other modalities besides not fulfilling the DSM-III-R dementia criteria, (2) functional activities of the person are mainly preserved or at least impairment is minimal, and (3) the person should have evidence of cognitive decline, either by self-assessment and/or the use of an informative report in conjunction with deficits on the objective cognitive tasks. Among participants without dementia, a Clinical Dementia Rating score of 0.5 was used as the objective cognitive impairment value to denote cognitive and functional impairment consistent with MCI.

### MRI analysis

Structural MRI studies were conducted using a 1.5- T system (ECHELON RX; Hitachi, Japan). Three-dimensional volumetric acquisition of T1-weighted turbo field echo images was conducted according to the brain MRI protocol for the Alzheimer’s Disease Neuroimaging Initiative (ADNI) study^26^ (Echo time/Repetition time, 4.0/9.2 ms; flip angle, 8°; Field of View, 240 mm; acquisition matrix, 192 × 192; number of slices, 170; voxel size, 0.9375 × 0.9375 mm; slice thickness, 1.2 mm). All T1-structural images were analyzed using FreeSurfer version 5.3 (http://surfer.nmr.mgh.harvard.edu)^27^ at Tohoku University and preprocessed according to the standard method. Volumes of the areas of interest were created by FreeSurfer^28^. The 37 areas were calculated as the sum of the volumes of the right and left sides for each area. The total brain volume (TBV) was calculated by summing the white and gray matter volumes. WMHs are lesions appearing as regions of decreased signal intensity on T1-weighted MRI images. They are considered manifestations of small vessel ischemic disease. The eTIV was used to normalize the volumetric value of 37 areas.

### Dental examination

Every patient underwent a thorough dental examination by a dental practitioner, according to the guidelines of the Third National Health and Nutrition Examination Survey^29^. Existing teeth were categorized as healthy, carious, or treated (incorporating crowned, inlay, and abutment teeth for prosthetic devices), including completely erupted third molars. Teeth not fully grown or congenitally absent, root remnants, and exceedingly mobile teeth were not counted.

### Nutritional status

We assessed nutritional status using a self-administered food frequency questionnaire (FFQ). This questionnaire has previously been validated and reported^30^. Each participant completed the questionnaire in advance, and trained dieticians checked it during the screening test. The average food intake per day was calculated using the frequency of meals per week and the amount of each food portion. The nutritional intake was determined using the Standard Tables of Food Composition in Japan (5th Revised Edition)^31^. All dietary nutrients were adjusted for total energy using the density method^32^.

### Statistical analysis

To compare participant characteristics, we used a one-way analysis of variance for mean calculations of continuous variables and a chi-square test for categorical variables. A two-tailed *p* < 0.05 indicates statistical significance. Analysis of covariance (ANCOVA) was employed to estimate and compare multivariable-adjusted values and their 95% confidence intervals for cognitive status, residual tooth number, and denture inclusion. In the multivariable-adjusted analysis, age, sex, educational level, hypertension, diabetes mellitus status, and LDL and HDL cholesterol levels were analyzed as covariates. Bonferroni adjustment was applied to multiple comparisons, with Bonferroni-corrected *p* < 0.05 considered statistically significant. The SPSS software suite (version 26; SPSS Inc., Chicago, IL, USA) was used for all statistical analyses.

Associations between dietary patterns and residual tooth number excluding denture in cognitively unimpaired individuals were evaluated using reduced rank regression (RRR) analysis, as previously described^16,33^. We selected nutrients that exhibited a significant decrease or increase in correlation tooth loss as the response variables. Consequently, dietary patterns were derived based on the intake of these nutrients across the food and beverage items. We employed the SAS procedure PLS (partial least squares) incorporating the options for the RRR analysis. Generally, principal component analysis uncovers combinations of predictors with substantial variance, neglecting response values. Conversely, multiple linear regression identifies a combination of predictors that optimally fits a response. RRR analysis amalgamates aspects of principal component analysis and multiple linear regression, extracting predictors that elucidate as much response variation as possible while maintaining considerable variance. For further information on alternative options and their SAS syntax, consult the chapter on The PLS Procedure in SAS/STAT 9.3 User’s Guide^34^. Before RRR analysis, all nutrients and food/beverage items were energy-adjusted using the density approach. In addition, we determined the explained percentages of variation and Pearson correlation coefficients between nutrient intake (response variables), food/beverage item consumption, and extracted dietary patterns.

### Reporting summary

Further information on research design is available in the Nature Research Reporting Summary linked to this article.

### Supplementary information

Reporting Summary

## Data Availability

The data compilations employed in the present investigation remain inaccessible to the public domain as they encompass sensitive medical information pertaining to the research subjects. However, the data are available on reasonable request and with the permission of the corresponding author.
