# Supplementary material for: Brain atrophy in normal older adult links tooth loss and diet changes to future cognitive decline
Source: NPJ Aging. 2024 Mar 22;10(1):20. doi: 10.1038/s41514-024-00146-4 (PMC10960014; doi:10.1038/s41514-024-00146-4)
Supplement: Supplementary file 1 — Reporting Summary [file 41514_2024_146_MOESM1_ESM.pdf]

Corresponding author(s): hiroyuki nakamura
Last updated by author(s): Mar 1, 2024

Reporting Summary

Nature Portfolio wishes to improve the reproducibility of the work that we publish. This form provides structure for consistency and transparency in reporting. For further information on Nature Portfolio policies, see our Editorial Policies and the Editorial Policy Checklist.

Statistics

For all statistical analyses, confirm that the following items are present in the figure legend, table legend, main text, or Methods section.

n/a	Confirmed	
☐	☒	The exact sample size (*n*) for each experimental group/condition, given as a discrete number and unit of measurement
☐	☒	A statement on whether measurements were taken from distinct samples or whether the same sample was measured repeatedly
☐	☒	The statistical test(s) used AND whether they are one- or two-sided<br>*Only common tests should be described solely by name; describe more complex techniques in the Methods section.*
☐	☒	A description of all covariates tested
☐	☒	A description of any assumptions or corrections, such as tests of normality and adjustment for multiple comparisons
☐	☒	A full description of the statistical parameters including central tendency (e.g. means) or other basic estimates (e.g. regression coefficient) AND variation (e.g. standard deviation) or associated estimates of uncertainty (e.g. confidence intervals)
☐	☒	For null hypothesis testing, the test statistic (e.g. F, t, r) with confidence intervals, effect sizes, degrees of freedom and P value noted<br>*Give P values as exact values whenever suitable.*
☒	☐	For Bayesian analysis, information on the choice of priors and Markov chain Monte Carlo settings
☒	☐	For hierarchical and complex designs, identification of the appropriate level for tests and full reporting of outcomes
☒	☐	Estimates of effect sizes (e.g. Cohen's *d*, Pearson's *r*), indicating how they were calculated

Our web collection on statistics for biologists contains articles on many of the points above.

Software and code

Policy information about availability of computer code

Data collection	N/A
Data analysis	N/A

For manuscripts utilizing custom algorithms or software that are central to the research but not yet described in published literature, software must be made available to editors and reviewers. We strongly encourage code deposition in a community repository (e.g. GitHub). See the Nature Portfolio guidelines for submitting code & software for further information.

Data

Policy information about availability of data

All manuscripts must include a data availability statement. This statement should provide the following information, where applicable:

- Accession codes, unique identifiers, or web links for publicly available datasets
- A description of any restrictions on data availability
- For clinical datasets or third party data, please ensure that the statement adheres to our policy

The data compilations employed in the present investigation remain inaccessible to the public domain as they encompass sensitive medical information pertaining to the research subjects. However, the data are available on reasonable request and with the permission of the corresponding author.

Research involving human participants, their data, or biological material

Policy information about studies with human participants or human data. See also policy information about sex, gender (identity/presentation), and sexual orientation and race, ethnicity and racism.

Reporting on sex and gender	This study was conducted on a population-based, longitudinal cohort of individuals within a specific age range over 60, both gender.
Reporting on race, ethnicity, or other socially relevant groupings	This report describes an ongoing study aiming to investigate cognitive deterioration in older adults Japanese individuals. Dementia screening was conducted on a population-based, longitudinal cohort of individuals within a specific age range. This study was conducted in Nakajima, situated in Nanao City within the Ishikawa Prefecture, Japan, and the research methodology has been previously described 16,24. From 2016 to 2018, 2,454 inhabitants aged ≥60 (representing 92.9% of the total demographics within this age bracket) participated in the study.
Population characteristics	see above
Recruitment	From 2016 to 2018, 2,454 inhabitants aged ≥60 (representing 92.9% of the total demographics within this age bracket) participated in the study.
Ethics oversight	Kanazawa University Medical Ethics Review Committee, Ryukyu University Medical Ethics Review Committee

Note that full information on the approval of the study protocol must also be provided in the manuscript.

Field-specific reporting

Please select the one below that is the best fit for your research. If you are not sure, read the appropriate sections before making your selection.

☐ Life sciences ☒ Behavioural & social sciences ☐ Ecological, evolutionary & environmental sciences

For a reference copy of the document with all sections, see nature.com/documents/nr-reporting-summary-flat.pdf

Behavioural & social sciences study design

All studies must disclose on these points even when the disclosure is negative.

Study description	population-based, longitudinal cohort of individuals
Research sample	From 2016 to 2018, 2,454 inhabitants aged ≥60 (representing 92.9% of the total demographics within this age bracket) participated in the study.
Sampling strategy	From 2016 to 2018, 2,454 inhabitants aged ≥60 (representing 92.9% of the total demographics within this age bracket) participated in the study.
Data collection	self-assessment and/or the use of an informative report in conjunction with deficits on the objective cognitive tasks. Structural MRI studies were conducted using a 1.5- T system. We assessed nutritional status using a self-administered food frequency questionnaire (FFQ).
Timing	From 2016 to 2018
Data exclusions	We excluded individuals who had no brain MRI examinations (n = 1,159), no dental examinations (N = 302), hemorrhagic and/or ischemic stroke lesions on MRI regardless of the presence or absence of neurological symptoms (n = 69), and no dietary history evaluations (N = 5). Stroke lesions (hemorrhagic and/or ischemic) were identified by two trained neuroradiologists blinded to the clinical information.
Non-participation	We excluded individuals who had no brain MRI examinations (n = 1,159), no dental examinations (N = 302), hemorrhagic and/or ischemic stroke lesions on MRI regardless of the presence or absence of neurological symptoms (n = 69), and no dietary history evaluations (N = 5). Stroke lesions (hemorrhagic and/or ischemic) were identified by two trained neuroradiologists blinded to the clinical information.
Randomization	From 2016 to 2018, 2,454 inhabitants aged ≥60 (representing 92.9% of the total demographics within this age bracket) participated in the study. We excluded individuals who had no brain MRI examinations (n = 1,159), no dental examinations (N = 302), hemorrhagic and/or ischemic stroke lesions on MRI regardless of the presence or absence of neurological symptoms (n = 69), and no dietary history evaluations (N = 5). Stroke lesions (hemorrhagic and/or ischemic) were identified by two trained neuroradiologists blinded to the clinical information.

Reporting for specific materials, systems and methods

We require information from authors about some types of materials, experimental systems and methods used in many studies. Here, indicate whether each material, system or method listed is relevant to your study. If you are not sure if a list item applies to your research, read the appropriate section before selecting a response.

Materials & experimental systems

n/a	Involved in the study
☒	☐ Antibodies
☒	☐ Eukaryotic cell lines
☒	☐ Palaeontology and archaeology
☒	☐ Animals and other organisms
☐	☒ Clinical data
☒	☐ Dual use research of concern
☒	☐ Plants

Methods

n/a	Involved in the study
☒	☐ ChIP-seq
☒	☐ Flow cytometry
☐	☒ MRI-based neuroimaging

Clinical data

Policy information about clinical studies

All manuscripts should comply with the ICMJE guidelines for publication of clinical research and a completed CONSORT checklist must be included with all submissions.

Clinical trial registration: *Provide the trial registration number from ClinicalTrials.gov or an equivalent agency.*

Study protocol: *Note where the full trial protocol can be accessed OR if not available, explain why.*

Data collection: *Describe the settings and locales of data collection, noting the time periods of recruitment and data collection.*

Outcomes: *Describe how you pre-defined primary and secondary outcome measures and how you assessed these measures.*

Plants

Seed stocks: *Report on the source of all seed stocks or other plant material used. If applicable, state the seed stock centre and catalogue number. If plant specimens were collected from the field, describe the collection location, date and sampling procedures.*

Novel plant genotypes: *Describe the methods by which all novel plant genotypes were produced. This includes those generated by transgenic approaches, gene editing, chemical/radiation-based mutagenesis and hybridization. For transgenic lines, describe the transformation method, the number of independent lines analyzed and the generation upon which experiments were performed. For gene-edited lines, describe the editor used, the endogenous sequence targeted for editing, the targeting guide RNA sequence (if applicable) and how the editor was applied.*

Authentication: *Describe any authentication procedures for each seed stock used or novel genotype generated. Describe any experiments used to assess the effect of a mutation and, where applicable, how potential secondary effects (e.g. second site T-DNA insertions, mosiacism, off-target gene editing) were examined.*

Magnetic resonance imaging

Experimental design

Design type: *Indicate task or resting state; event-related or block design.*

Design specifications: *Specify the number of blocks, trials or experimental units per session and/or subject, and specify the length of each trial or block (if trials are blocked) and interval between trials.*

Behavioral performance measures: *State number and/or type of variables recorded (e.g. correct button press, response time) and what statistics were used to establish that the subjects were performing the task as expected (e.g. mean, range, and/or standard deviation across subjects).*

Acquisition

Imaging type(s): Structural

Field strength: 1.5- T

Sequence & imaging parameters: Echo time/Repetition time, 4.0/9.2 ms; flip angle, 8°; Field of View, 240 mm; acquisition matrix, 192 × 192; number of slices, 170; voxel size, 0.9375 × 0.9375 mm; slice thickness, 1.2 mm

Area of acquisition: whole brain scan

Diffusion MRI ☐ Used ☒ Not used

Preprocessing

Preprocessing software: FreeSurfer version 5.3

Normalization: estimated total intracranial volume (eTIV) was used to normalize the volumetric value

Normalization template: estimated total intracranial volume (eTIV) was used to normalize the volumetric value

Noise and artifact removal: *Describe your procedure(s) for artifact and structured noise removal, specifying motion parameters, tissue signals and physiological signals (heart rate, respiration).*

Volume censoring: FreeSurfer version 5.3

Statistical modeling & inference

Model type and settings: *Specify type (mass univariate, multivariate, RSA, predictive, etc.) and describe essential details of the model at the first and second levels (e.g. fixed, random or mixed effects; drift or auto-correlation).*

Effect(s) tested: ANCOVA

Specify type of analysis: ☒ Whole brain ☐ ROI-based ☐ Both

Statistic type for inference (See Eklund et al. 2016): *Specify voxel-wise or cluster-wise and report all relevant parameters for cluster-wise methods.*

Correction: Bonferroni-corrected

Models & analysis

n/a	Involved in the study
☒	☐ Functional and/or effective connectivity
☒	☐ Graph analysis
☐	☒ Multivariate modeling or predictive analysis

Multivariate modeling and predictive analysis: Analysis of covariance (ANCOVA) was employed to estimate and compare multivariable-adjusted values
